# Virtual Reality to Enhance Understanding of Congenital Heart Disease

**DOI:** 10.3390/jcdd12120495

**Published:** 2025-12-15

**Authors:** Shanti L. Narasimhan, Ali H. Mashadi, Syed Murfad Peer, Kishore R. Raja, Pranava Sinha, Satoshi Miyairi, Juan Carlos Samayoa Escobar, Devin Chetan, Yu-Hui Huang, Paul A. Iaizzo

**Affiliations:** 1 Department of Pediatrics, University of Minnesota Masonic Children’s Hospital, Minneapolis, MN 55454, USA; ahmashadi01@gmail.com (A.H.M.); raja0161@umn.edu (K.R.R.); chetan@umn.edu (D.C.); 2 Department of Cardiothoracic Surgery, University of Minnesota Masonic Children’s Hospital, Minneapolis, MN 55454, USA; smpeer@umn.edu (S.M.P.); sinha228@umn.edu (P.S.); miyai003@umn.edu (S.M.); 3 Department of Radiology, University of Minnesota, Minneapolis, MN 55454, USA; huan2098@umn.edu; 4 Visible Heart^®^ Laboratories, Department of Surgery, The Institute for Engineering in Medicine, University of Minnesota, Minneapolis, MN 55455, USA; iaizz001@umn.edu

**Keywords:** virtual reality, congenital heart disease, education, families

## Abstract

This retrospective study evaluated the clinical utility of Virtual Reality (VR) in visualizing extracardiac CHD (eCHD) abnormalities involving great vessels, pericardium, or structures outside the heart in nine pediatric patients. Anonymized computed tomography angiography (CTA) DICOM images were processed using Elucis (Version 1.10 elucis next) software to generate interactive 3D models via segmentation. VR models were reviewed for a variety of cases: vascular rings (two with right aortic arch, aberrant left subclavian artery, and diverticulum of Kommerell; two with double aortic arch), pericardial teratomas (*n* = 2), right superior vena cava draining into the left atrium (*n* = 1), left pulmonary artery sling (*n* = 1), and aortopulmonary window (*n* = 1). VR video images were presented during weekly heart center conferences. A survey conducted among heart center staff assessed the perceived value of VR in clinical practice. A total of 62% found traditional diagnostic modalities very effective, 100% considered VR a valuable diagnostic tool, 65% responded positively to VR image resolution, 50% highlighted its educational benefit, 81% believed VR enhanced diagnostic accuracy and surgical planning, and 100% would recommend its use to colleagues. This study demonstrates the successful integration of VR-based segmentation into clinical workflows, underlining its potential as both an educational resource and a tool to support diagnostic and surgical decision-making.

## 1. Introduction

Congenital heart disease (CHD) can be broadly categorized into intracardiac and extracardiac lesions, with a general incidence of approximately 3 to 8 per 1000 live births. Extracardiac CHD (eCHD) refers to structural abnormalities involving the great vessels, pericardium, or other structures located outside the heart’s four chambers. Common examples of these include vascular rings, double aortic arch (DAA), left pulmonary artery (LPA) sling, intrapericardial teratoma, aortopulmonary (AP) window, and drainage of the superior vena cava (SVC) into the left atrium (LA). A wide spectrum of clinical variation exists in eCHD, with subtle but important variations accounting for timing of presentation throughout childhood. When reviewing two-dimensional imaging, it can be challenging to fully appreciate the complexity of the geometric relationships underpinning the 3D cardiac anatomy. Advancements in Virtual Reality (VR) segmentation methodologies applied to the cardiac field have introduced innovative solutions to address previous known limitations. VR segmentation transforms 2D imaging into immersive 3D models, facilitating improved visualization of complex anomalies for preoperative planning and real-time manipulation of patient-specific imaging data [1].

VR demonstrates significant value not only in interventional planning for eCHD, but also as a tool for knowledge transfer—both to our heart center team and to trainees. This case series highlights several impactful applications of VR segmentation in eCHD, particularly in enhancing collaboration and supporting shared decision-making within the heart center team.

## 2. Materials and Methods

This is a single-center retrospective study of nine children diagnosed with eCHD at the University of Minnesota between 2023 and 2024. Our cohort included four with vascular rings, two with chest masses, and one each with right SVC to LA, LPA sling, and AP window.

This study was approved by the University of Minnesota Institutional Review Board, in December 2024, MOD00052087.

### 2.1. Protocol

When a patient presented with eCHD, relevant imaging modalities, including transthoracic echocardiography (TTE), cardiac computed tomography angiography (CTA), and 3D-printed models, were reviewed. For surgical planning, high-resolution CT images were obtained in DICOM format. These images were first de-identified to ensure patient confidentiality and then securely compiled on an encrypted storage device. The DICOM datasets were imported into Elucis VR Software (Realize Medical, Ottawa, ON, Canada), a specialized platform for immersive visualization and segmentation of complex cardiac anatomies.

Within Elucis VR, semi-automated segmentation tools were used to delineate cardiac structures. Initial segmentation involved thresholding techniques to differentiate cardiac tissues from surrounding structures based on radiodensity values. Manual refinement was performed using VR controllers to correct any inaccuracies, such as delineating thin myocardial walls, valve leaflets, or complex congenital defects that were not adequately captured by automated methods.

Segmentation focused on key anatomical components relevant to surgical planning, including the atria, ventricles, great vessels, septal defects, and associated anomalous vessels. Each segmented structure was carefully reviewed and validated by a multidisciplinary team, including cardiac radiologists and congenital heart surgeons, to ensure anatomical fidelity.

Once segmentation was completed, a 3D virtual replica was generated, allowing interactive manipulation within the VR environment. This model provided detailed spatial understanding of each patient’s unique cardiac anatomy, facilitating preoperative planning and surgical rehearsal.

For surgical planning, CT images obtained from DICOM images were de-identified, compiled onto a secure storage disk, and imported into Elucis VR Software (Realized Medical, Ottawa, ON, Canada) for segmentation to generate a 3D replica of each patient’s unique cardiac anatomies. After TTE, CT, and 3D models were presented in the conference, the 3D VR segmentation video recordings were displayed and shared with the heart center team during our weekly surgical/interventional conference. The evaluation followed a multi-stage approach:

Exposure Stage: Providers were introduced to VR technology at a separate time, and the VR images that were segmented were presented during the weekly surgical/interventional heart team conference.

Interaction Stage: Participants had the opportunity to explore VR reconstructions of patient-specific cardiac anatomy either individually or in small groups.

Comparison Stage: Respondents were asked to reflect on and compare the utility of VR to traditional diagnostic modalities (e.g., echocardiography, CT/MRI, catheterization).

Feedback Stage: A post-experience online survey was distributed to capture impressions across domains such as diagnostic clarity, planning confidence, and educational value.

### 2.2. Survey

A structured, 7-item survey was conducted among clinical staff members at a heart center to assess the perceived value of Virtual Reality technology in congenital heart disease diagnosis and interventional planning. The survey was administered electronically using Google Forms, enabling efficient data collection and quantitative analysis through a centralized, shared database, to the providers immediately after the weekly surgical/interventional heart center team conference. Participation was voluntary and the survey was anonymous. Of the 40 providers invited to participate, 26 completed the survey (response rate: 65%). Respondents included 3 cardiac surgeons, 8 cardiologists, 5 cardiology fellows, 3 advanced practice providers/nurse practitioners (APPs/NPs), 2 catheterization laboratory staff, 2 intensive care unit (ICU) physicians, 1 anesthesiologist, and 2 perfusionists. Four possible answers were available for each question, detailing the provider’s impression of the utility of VR. Descriptive statistics were then used to summarize survey results. The survey was composed of the following questions:

1. What is your current role?
2. Which of the following diagnostic modalities have you used in your practice? (Select all that apply).
  - Echocardiography (Echo)
  - CT/MRI
  - Cardiac catheterization (Cath)
  - Virtual Reality
  - Other
3. How effective do you find the traditional diagnostic methods (i.e., Echo, CT/MRI, Cath) to be in diagnosing CHD?
  - Very effective
  - Effective
  - Neutral
  - Not effective
4. After experiencing Virtual Reality technology, how would you rate its effectiveness in diagnosing and planning for interventions in CHD compared to traditional modalities?
  - Much more effective
  - Somewhat effective
  - No change
  - Not effective
5. What aspect of Virtual Reality did you find most beneficial for CHD?
  - Diagnostic tool integration
  - Educational tool
  - Image resolution
  - Other
6. How likely is it that you would recommend Virtual Reality to a friend or colleague?
  - Likely
  - Unlikely
  - Other
7. Please share any additional suggestions or comments that you have regarding Virtual Reality.

## 3. Results

Each imaging modality has its limitations; however, a multimodality imaging approach provides a more comprehensive evaluation of the surrounding structures.

### 3.1. Case Presentations

#### 3.1.1. Case 1

**Figure 1:**
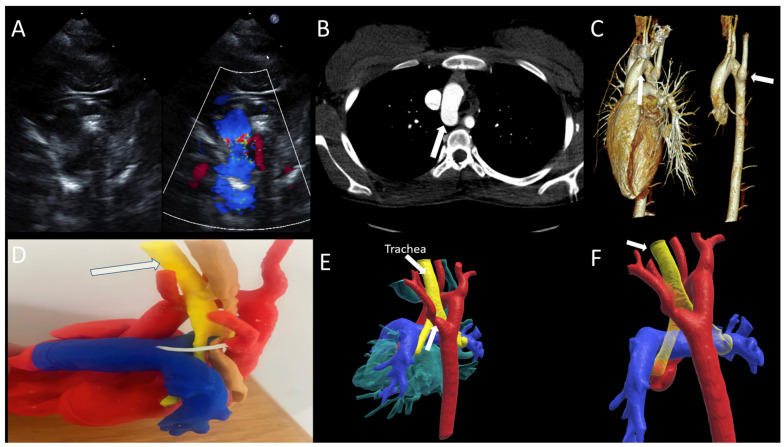
(**A**) Two-dimensional and Color Doppler TTE demonstrating RAA with aberrant left subclavian artery; (**B**,**C**) CT scan highlighting RAA from anterior and lateral views identifying the vessel contributing to airway compression; (**D**) 3D print; (**E**,**F**) VR reconstruction detailing the trachea in yellow and RAA.

A term infant with 22q11 deletion syndrome underwent postnatal TTE that revealed a right aortic arch (RAA) with an aberrant left subclavian artery (Figure 1A). CTA highlights the RAA from anterior and lateral views, identifying the vessel contributing to airway compression and diverticulum of Kommerell (Figure 1B,C). Three-dimensional printing illustrates the esophagus and trachea encircled within the vascular ring (Figure 1D). VR imaging clearly demonstrates the airway anatomy in yellow and the contribution of the diverticulum of Kommerell to the formation of the ring (Figure 1E,F). Based upon this assessment, the patient underwent division of the ligamentum arteriosum, translocation of the left subclavian artery, and resection of the diverticulum of Kommerell via a left lateral thoracotomy. He recovered well and remains in good condition.

#### 3.1.2. Case 2

**Figure 2:**
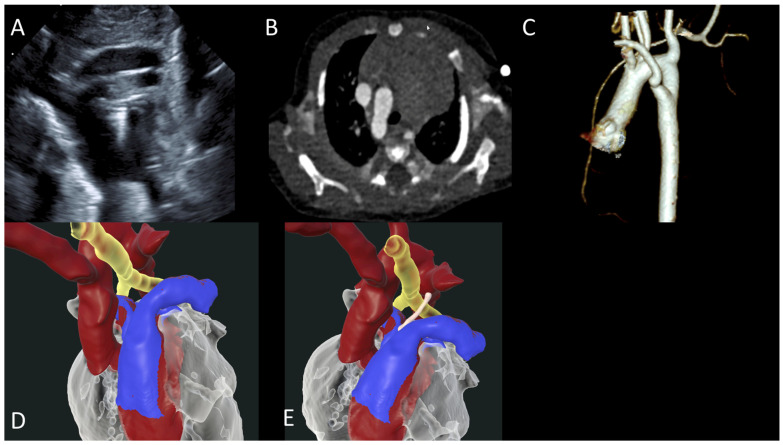
(**A**) TTE showing RAA; (**B**,**C**) CT indicating RAA with aberrant left subclavian artery compression of the trachea; (**D**,**E**) VR models demonstrating the spatial relationship between aorta (red), trachea (yellow), pulmonary artery (blue) and white represents ligamentum arteriosus completing the ring.

A term infant with PHACES syndrome was prenatally diagnosed with a RAA, left ductus arteriosus, and an aberrant right subclavian artery, consistent with a vascular ring. Postnatal TTE confirmed the diagnosis (Figure 2A). CTA provides clearer delineation of the diverticulum of Kommerell (Figure 2B,C). VR models demonstrate the spatial relationship between aorta (red), trachea (yellow), and pulmonary artery (blue), and the ligamentum arteriosus is rendered in white, completing the ring (Figure 2D,E). This comprehensive imaging assessment enabled a successful elective surgical intervention at 8 months of age, including ligation and division of the left ligamentum arteriosum, resection of the diverticulum of Kommerell, and translocation of the aberrant retroesophageal left subclavian artery to the left common carotid artery via a left thoracotomy. The patient had an uncomplicated postoperative course.

#### 3.1.3. Case 3

**Figure 3:**
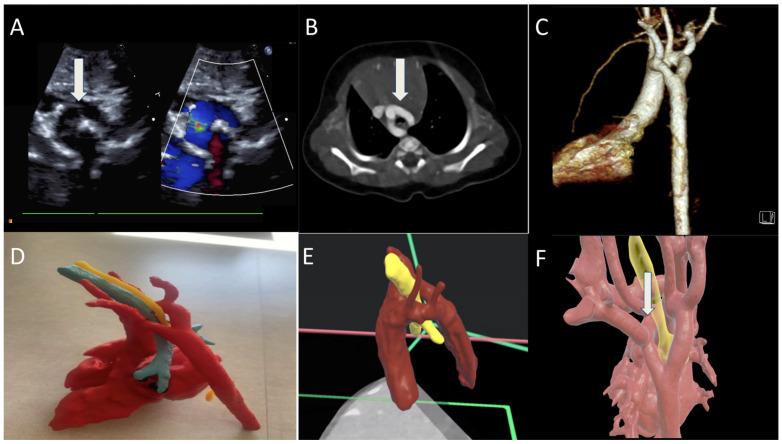
(**A**) TTE 2D with color showing double aortic arch around the airway; (**B**,**C**) CT of chest with double aortic arch encircling the trachea; (**D**) 3D-printed model of the DAA (red), teal is trachea, and yellow is the esophagus; (**E**,**F**) VR models detailing the double arch (red) and trachea (yellow) encircled by the vascular anomaly.

An infant had a prenatal diagnosis of DAA. Postnatal TTE confirmed DAA and left sided patent ductus arteriosus (Figure 3A). CTA showed a dominant right arch larger than the left aortic arch (Figure 3B,C). Three-dimensional printing illustrates the esophagus and trachea encircled by the DAA (Figure 3D). VR imaging provides a clear depiction of the airway anatomy encircled by the DAA, with color-coded anatomical structures facilitating improved spatial orientation and understanding (Figure 3E,F). At 2 months of age, the patient developed viral bronchiolitis, which was complicated by respiratory distress requiring mechanical ventilation. At 2.5 months, he underwent vascular ring division involving resection of the left aortic arch and division of the ligamentum arteriosum via a left thoracotomy. Subsequent laryngoscopy revealed a normal upper airway but severe distal tracheal collapse extending to the carina. CTA demonstrated significant narrowing of the distal trachea just above the carina. He therefore underwent an aortic arch uncrossing through a median sternotomy. A tracheostomy was performed at 5 months of age due to persistent airway obstruction.

#### 3.1.4. Case 4

**Figure 4:**
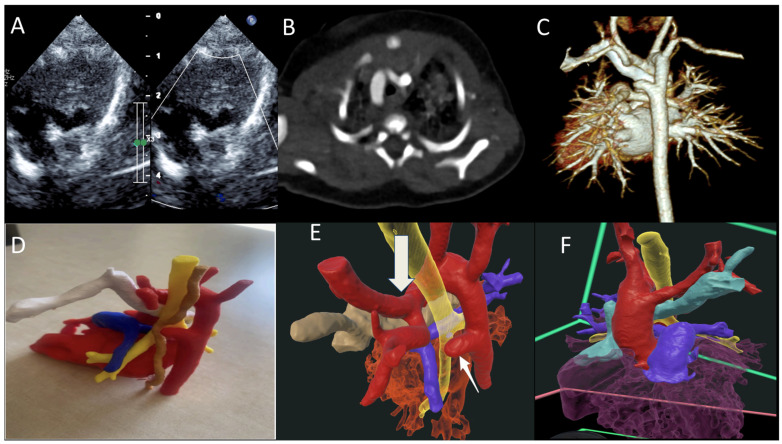
(**A**) TTE showing double aortic arch with dominant right arch arrow (DAA); (**B**,**C**) CT scan of the DAA with dominant right aortic arch, Kommerell diverticulum and atretic left arch; (**D**) 3D-printed model trachea (yellow), esophagus (brown), retro-aortic innominate vein (white); (**E**,**F**) VR showing DAA (red), trachea (yellow), retro-aortic innominate vein (teal) ring, completed by the atretic left aortic arch, Kommerell diverticulum (arrow).

An infant was found to have DAA during workup for a heart murmur during a hospitalization for viral bronchiolitis. TTE revealed DAA with a dominant right arch (Figure 4A). CTA confirmed this diagnosis, in addition to showing a retro-aortic innominate vein, Kommerell diverticulum, and atretic left arch, forming a vascular ring (Figure 4B,C). Three-dimensional printing illustrated the encirclement of the airway and esophagus within the vascular ring (Figure 4D). VR showed DAA (red), trachea (yellow), and retro-aortic innominate vein (teal) ring, which was completed by an atretic left aortic arch, Kommerell diverticulum (Figure 4E,F). To date, the child has remained asymptomatic, and therefore the patient’s family has opted to wait until the presentation of symptoms for surgical repair.

#### 3.1.5. Case 5

**Figure 5:**
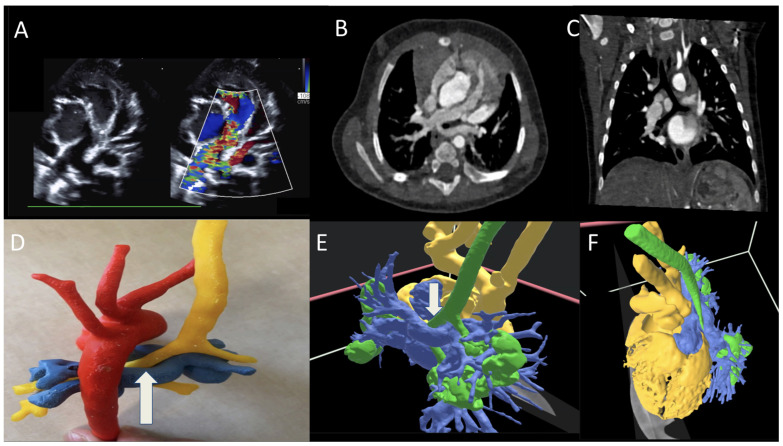
(**A**) shows an 2D and color echocardiogram of the LPA sling; (**B**,**C**) are CT; (**D**) is a 3D-printed model of the LPA sling with trachea in yellow which is narrowed. Tracheal bronchus. Accessory bronchus originates directly from the trachea; (**E**,**F**) are VR images with an arrow pointing towards the LPA sling and trachea in green.

An infant was diagnosed with Tetralogy of Fallot and an LPA sling. At 3 months of age, the child began developing severe pulmonary stenosis. TTE showed that the LPA arose from the right pulmonary artery with a peak gradient of 37 mm Hg for the LPA and 26 mm Hg for the right pulmonary artery (Figure 5A). CTA confirmed the presence of the sling and narrowing of the trachea with tracheal bronchus and an accessory bronchus originating directly from the trachea (Figure 5B,C). The 3D-printed model showed an LPA sling, as well as narrowing of the trachea with tracheal bronchus and an accessory bronchus originating directly from the trachea (Figure 5D). VR segmentation enabled for better visualization of the sling and the airway compression (Figure 5E,F). He subsequently underwent surgical repair of Tetralogy of Fallot with a transannular patch, with the LPA being reimplanted to normal anatomic position. At 7 months of age, the child underwent cardiac catheterization and stenting of the LPA.

#### 3.1.6. Case 6

**Figure 6:**
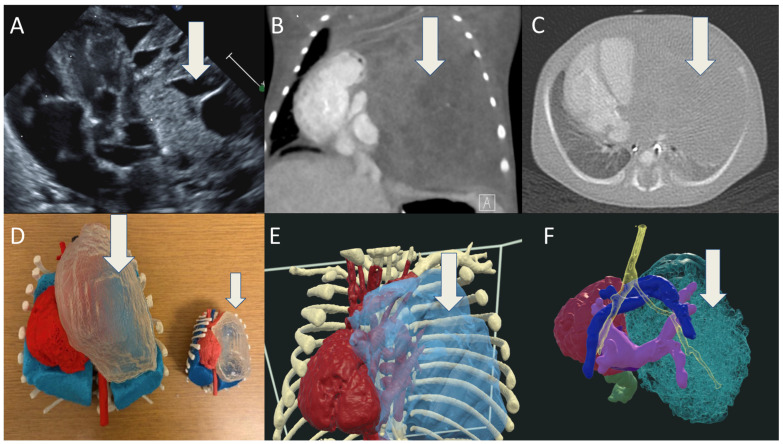
(**A**) TTE showing the teratoma (arrow) and the heart on the right chest; (**B**,**C**) CT chest revealing the multicystic mass in the left chest and displacement of the heart into the right thorax; (**D**) is a 3D printed model of the teratoma; (**E**,**F**) VR segmentation of the teratoma and narrowing of the left bronchus, arrow.

This patient was a male with fetal diagnosis of an intrapericardial mass with fetal hydrops at 29 weeks of gestational age who subsequently underwent fetal pericardiocentesis. This infant was delivered at 33 5/7 weeks of gestation. He was in respiratory failure and required mechanical conventional ventilation immediately after birth and remained that way until surgery. A rigid bronchoscopy showed significant posterior compression down to the carina. His TTE revealed a large polycystic mass in the left anterior chest with dextroposition (Figure 6A). CTA revealed a large cystic mass and solid left chest/mediastinal mass with significant mass effect and LPA hypoplasia (Figure 6B,C). Three-dimensional modeling confirmed its location in the left chest and the vast size of the mass (Figure 6D). VR showed that the mass was along the great vessels—notably the LPA, ascending aorta, and the left heart. VR was utilized to map the tumor’s connections, and it was believed that there was no cardiac involvement, including no connection to the pulmonary veins. Significant displacement of the tracheobronchial tree was also noted with the most severe being narrowing of/at the left main stem bronchus (Figure 6E,F). Thus, he underwent surgical resection of the mass at 2 weeks of age with cardiopulmonary bypass on standby. Intraoperative evaluation revealed that the tumor occupied the entire left hemithorax and anterior mediastinum, and the tumor pedicles were attached to the right lateral aspect of the ascending aorta about 3–4 mm above the sinotubular junction. Attempted manipulation of the mass resulted in the loss of arterial pressure, and therefore cardiopulmonary bypass was initiated, and the tumor was successfully excised in its entirety. Pathologic examination of the specimen revealed an immature teratoma.

#### 3.1.7. Case 7

**Figure 7:**
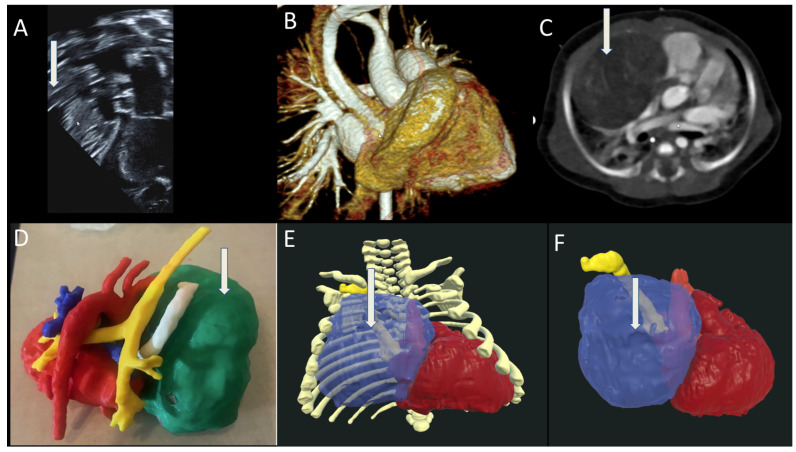
(**A**) TTE 2D showing teratoma on the right side of the chest, arrow; (**B**,**C**) chest CT revealing the teratoma in the right chest with close proximity to the SVC and the ascending aorta, leftward displacement of the heart; (**D**) 3D print; (**E**,**F**) VR segments of the teratoma in blue, SVC in yellow, heart in red.

An ex-27 weeks premature infant with a birth weight of 1259 g was transferred from an outside hospital at 34 weeks due to a large mediastinal mass occupying the right hemithorax. The 2D TTE showed a mass that occupied the right side of the chest (Figure 7A). CTA showed a large mediastinal mass extending into the right chest measuring 5.4 × 3.5 cm. The mass displaced the heart to the left and the SVC and right pulmonary vessels posteriorly. The trachea was mildly displaced to the right with mild anterior posterior narrowing of the carina (Figure 7B,C). The 3D model showed the size of the mass clearly, with no cardiac involvement (Figure 7D). VR showed that the mass caused compression of the SVC in yellow (Figure 7E,F). Intraoperatively, the mass was found to be adherent to the superior aspect of the anterior pericardial wall, with intrapericardial extension into the adventitia of the ascending aorta 3–4 mm inferior to the innominate artery origin. The chest mass was consistent with an immature teratoma. Resection was successful and he has been doing well in the post operative period.

#### 3.1.8. Case 8

**Figure 8:**
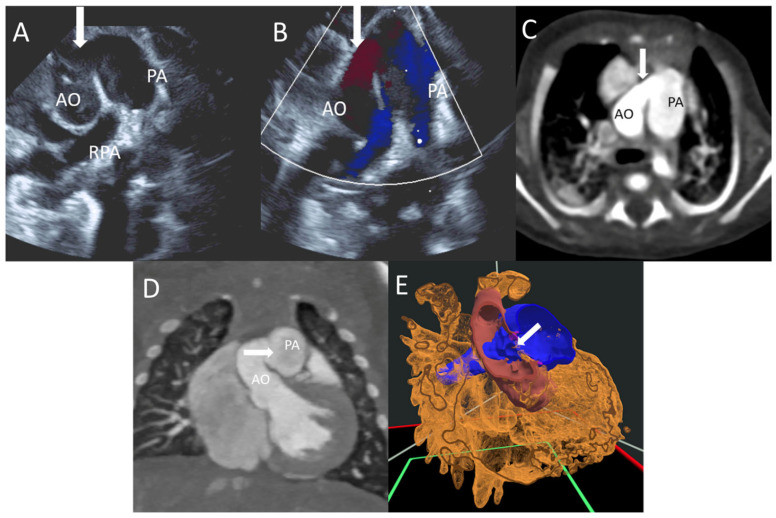
(**A**,**B**) TTE 2D and color of the AP window arrow; (**C**,**D**) CT imaging of the AP window; (**E**) VR segmentation with the arrow pointing the AO and PA connection.

A newborn at 34 2/7 weeks of gestation age presented with respiratory distress, hypoglycemia, and thrombocytopenia. TTE demonstrated AP window (Figure 8A,B). CTA showed type 1 AP window. The lesion was 3 mm in length and 3–4 mm in diameter, being located just above the sinus of Valsalva, a few millimeters above the semilunar valve (Figure 8C,D). VR further revealed the aortic-to-pulmonary communication (Figure 8E). He underwent surgical repair of the type 1 AP window at 3 weeks of age.

#### 3.1.9. Case 9

**Figure 9:**
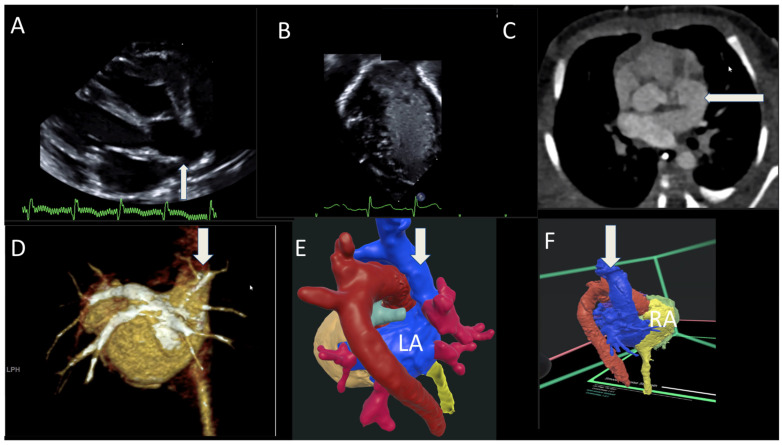
(**A**,**B**) TTE 2D and bubble contrast of the SVC connection to the LA arrow; (**C**,**D**) CT chest showing SVC draining into the LA; (**E**,**F**) VR showing the SVC anomalous draining into the LA. RA—right atrium. LA—left atrium.

A newborn infant had both a TTE and bubble study which showed aberrant drainage of the right SVC to the LA during a workup for ongoing hypoxia (Figure 9A,B). CTA showed SVC draining into the LA along with partial anomalous pulmonary venous return, consisting of right upper and middle pulmonary veins draining into the posterolateral SVC (Figure 9C,D). VR showed the SVC anomalously draining into the LA with color-coded structures, which improved anatomical orientation and understanding (Figure 9E,F). The infant underwent surgical correction of her SVC and right upper/middle pulmonary veins at 2 months of age, and she has been doing well.

### 3.2. Survey

A survey was conducted with members of the heart center team amidst the integration of VR to evaluate its utility in diagnostic and educational applications. A total of 40 participants were invited to complete the survey, with 26 completing it (response rate: 65%). Items 3–6 (A–D) from the 7-item survey were collected for data analysis.

Survey responses demonstrated the following perceptions of VR and traditional imaOKging modalities:

(A) Effectiveness of traditional diagnostic modalities: Regarding conventional imaging methods such as echocardiography, cardiac CT angiography (CTA), magnetic resonance imaging (MRI), and cardiac catheterization, 62% (16/26) rated these modalities as “very effective,” while 38% (10/26) rated them as “effective.”
(B) Improved diagnostic and surgical planning: A total of 81% (21/26) of participants believed that VR enhanced the effectiveness of diagnostic evaluation and surgical planning compared to traditional imaging methods, while 19% (5/26) rated them as somewhat effective.
(C) Beneficial aspects of VR: All respondents (100%, 26/26) considered VR a valuable diagnostic tool in the clinical setting. A total of 65% (17/26) of respondents rated the image resolution of VR models positively, indicating technological maturity sufficient for clinical interpretation. Half of the respondents (50%, 13/26) acknowledged the educational benefits of VR, suggesting potential underutilization in teaching environments.
(D) Willingness to recommend: All respondents (100%, 26/26) indicated they would recommend VR technology to colleagues.

Figure 10.

Image resolution: A total of 65% (17/26) of respondents rated the image resolution positively.

Diagnostic value of VR: A total of 100% (26/26) of respondents considered VR a valuable diagnostic tool.

Educational value: A total of 50% (13/26) acknowledged VR’s educational value.

Willingness to recommend: A total of 100% (26/26) stated they would recommend VR to colleagues.

Improved diagnostic and surgical planning: A total of 81% (21/26) believed VR enhanced diagnostic and surgical planning effectiveness compared to traditional imaging methods.

Effectiveness of traditional diagnostic modalities: Echocardiography, cardiac CTA, magnetic resonance imaging (MRI), cardiac catheterization.

A total of 38% (10/26) rated them as effective.

A total of 62% (16/26) rated them as very effective.

## 4. Discussion

Vascular ring, LPA sling, AP window, anomalous drainage of the right SVC into the LA, and cardiac tumors are complex lesions and often present with significant variability, making accurate diagnosis or complete characterization challenging [2,3,4,5,6,7,8]. Therefore, integration of multimodality imaging into the clinical workflow for precise diagnoses and management is invaluable. Our early experience suggests that integration of VR with medical imaging of CHD patients, offers significant advancements in both diagnosis and education of the care team and patient families [9,10,11,12,13]. In our cohort study, VR was incorporated alongside traditional imaging modalities such as echocardiography, CTA, cardiac MRI, 3D-printed models, and cardiac catheterization during the weekly heart center conferences. These uses of VR were found to enhance multidisciplinary team discussions, improve diagnostic confidence, and facilitate collaborative decision-making.

The survey results demonstrate a strong endorsement of VR as a diagnostic and planning tool among participants, with unanimous positive agreement on its value in clinical settings. Notably, 81% of respondents indicated that VR not only enhances diagnostic accuracy but also improves preoperative surgical planning when compared to traditional imaging modalities alone. These findings suggest that VR technology provides a more intuitive and immersive understanding of complex anatomical structures/relationships, which may facilitate better clinical decision-making. The positive reception of image resolution by 65% of participants underscores the technological maturity of VR platforms. While not universally praised, this majority indicates that the current state of VR is sufficient for meaningful clinical interpretation. However, this also highlights an area for further development, particularly in refining resolution and rendering support to meet the clinical expectations of all stakeholders. Interestingly, only 50% of participants recognized the educational benefits of VR, suggesting possible underutilization of this modality in teaching environments. This could reflect either a lack of exposure to VR-based educational content or a gap in VR training modules tailored to medical curricula. VR remains a relatively novel technology in many clinical settings, and its role in formal medical education has not yet been fully established. Learners are often first expected to develop a strong foundation in conventional imaging modalities—such as echocardiography, cardiac catheterization, CTA and MRI—which remain the standard of care. As such, VR may be viewed by some as an adjunct rather than a core instructional method, especially early in training. Additionally, the effectiveness of any educational tool depends not just on the technology itself but also on how it is integrated into curricula. Some users might have encountered VR in a more exploratory or unstructured context, which could limit its perceived educational value. Another possible explanation is that educational outcomes in medicine are often assessed through standardized assessments and traditional competencies. Since VR-based education is still evolving, its direct impact on measurable outcomes may not yet be clear to educators or learners. Therefore, while the technology is promising, its educational value might not be immediately apparent without further institutional support, curricular integration, and evidence-based validation.

Further exploration into the design and implementation of VR educational tools may help bridge this gap. Importantly, 100% of respondents stated they would recommend VR to their colleagues. This unanimous support reflects both satisfaction with the technology and confidence in its utility and could reflect a broader readiness within the medical community to adopt VR into routine practice. In contrast, traditional imaging methods were deemed merely “effective” by a minority (38%), whereas 62% described them as “very effective.” While this suggests that conventional techniques still hold value, especially among experienced clinicians familiar with their interpretation, it also implies a growing awareness of their limitations—particularly in terms of spatial comprehension and interactive engagement, which VR effectively addresses. Overall, these findings support the integration of VR into diagnostic and planning workflows, with potential benefits extending into surgical decision-making and interdisciplinary communication. Nevertheless, further studies involving larger and more diverse participant pools are needed to validate these early findings and assess the long-term impact of VR in clinical practice. The uses of VR we described here demonstrated value in improving visualization of complex anatomical relationships, such as airway and vascular anatomies, which proved particularly beneficial in complex teratoma cases. This technology facilitated clearer communication with the heart center team and enabled enhanced collaborations among the surgical team. VR’s interactive capabilities, including real-time manipulations and annotations, were an improvement over traditional 2D imaging methods, which often required mental reconstructions of the presented anatomy [9,10,11,12,13].

Looking towards the future, our experience has led us to believe that the ability to transform 2D imaging into high-resolution interactive VR models, may enable better understanding of these complex clinical scenarios to patients and their families.

### Limitations

While our report highlights the benefits of employing VR technologies in eCHD clinical evaluations and treatment planning, several limitations exist. This study focused only on patients with imaging suitable for VR segmentation. This may exclude more complex cases, limiting generalizability. Furthermore, the small sample size reported restricts the statistical significance of the study and does not capture the full spectrum of variability in CHD presentations.

Though utilizing a single VR software platform (Elucis) helps maintain controlled data management, this reliance may also introduce technological constraints. Feedback on the utility of VR was primarily collected through subjective surveys, which introduces potential bias and limits the objectivity of the evaluation. The collaborative capabilities remain limited, notably when only one provider wears the VR headset while others view the images through a 2D panel, potentially reducing the collective interpretative experience.

Additionally, the integration of VR into clinical practice faces several intrinsic challenges. The steep learning curve and requisite technological proficiency may hinder rapid adoption among clinicians less familiar with VR systems. The process of segmentation Citself remains time-consuming and labor-intensive, which may impact workflow efficiency. Another critical limitation is the lack of tactile feedback during navigation of VR models, reducing the sensory experience that can be crucial for understanding complex cardiac anatomy.

Finally, the resource-intensive nature of VR—demanding specialized hardware, software, and trained personnel—may impede widespread implementation across diverse healthcare settings, particularly in resource-limited environments.

## 5. Conclusions

This study demonstrates the successful integration of VR-based segmentation into clinical workflows as a promising tool in the diagnosis, education, and surgical planning of eCHD. Its integration with existing imaging technologies could greatly enhance patient care and training, especially in such complex pediatric cases. The positive feedback we received underscores the potential for VR to improve both clinical outcomes and educational experiences for healthcare providers.

## Figures and Tables

**Figure 10:**
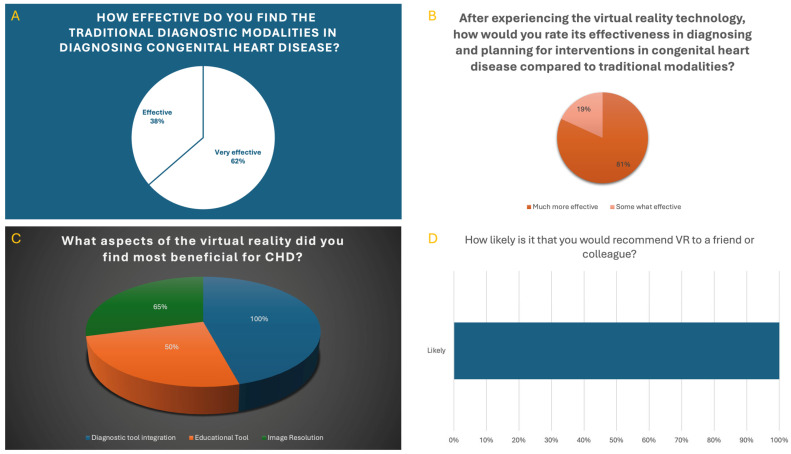
(**A**–**D**) show study survey results from the heart center team.

## Data Availability

Data may be made available upon request to the corresponding author.

## Abbreviations

The following abbreviations are used in this manuscript:

AP	Aortopulmonary
CHD	Congenital Heart Disease
CTA	Computed Tomography Angiography
DAA	Double Aortic Arch
eCHD	Extracardiac Congenital Heart Disease
LA	Left Atrium
LPA	Left Pulmonary Artery
MRI	Magnetic Resonance Imaging
SVC	Superior Vena Cava
TTE	Transthoracic Echocardiography
VR	Virtual Reality

## References

[1] Raimondi F., Vida V., Godard C., Bertelli F., Reffo E., Boddaert N., El Beheiry M., Masson J.B. (2021). Fast-track virtual reality for cardiac imaging in congenital heart disease. J. Card. Surg..

[2] Hastreiter A.R., D’Cruz I.A., Cantez T., Namin E.P., Licata R. (1966). Right-sided aorta. I. Occurrence of right aortic arch in various types of congenital heart disease. II. Right aortic arch, right descending aorta, and associated anomalies. Br. Heart J..

[3] Lowe G.M., Donaldson J.S., Backer C.L. (1991). Vascular rings: 10-year review of imaging. Radiographics.

[4] KRahmath M.R., Durward A. (2023). Pulmonary artery sling: An overview. Pediatr. Pulmonol..

[5] Jansen C., Hruda J., Rammeloo L., Ottenkamp J., Hazekamp M.G. (2006). Surgical repair of aortopulmonary window: Thirty-seven years of experience. Pediatr. Cardiol..

[6] Jacobs J.P., Quintessenza J.A., Gaynor J.W., Burke R.P., Mavroudis C. (2000). Congenital Heart Surgery Nomenclature and Database Project: Aortopulmonary window. Ann. Thorac. Surg..

[7] Baggett C., Skeen S.J., Gantt D.S., Trotter B.R., Birkemeier K.L. (2009). Isolated right superior vena cava drainage into the left atrium diagnosed noninvasively in the peripartum period. Tex. Heart Inst. J..

[8] Singh V., Kakkar S., Arora A., Garg A., Harjai M.M. (2015). A rare case of intra-pericardial teratoma presenting as a mediastinal mass in an infant. Med. J. Armed Forces India.

[9] Lan L., Mao R.Q., Qiu R.Y., Kay J., de Sa D. (2023). Immersive Virtual Reality for Patient-Specific Preoperative Planning: A Systematic Review. Surg. Innov..

[10] Awori J., Friedman S.D., Howard C., Kronmal R., Buddhe S. (2023). Comparative effectiveness of virtual reality (VR) vs 3D printed models of congenital heart disease in resident and nurse practitioner educational experience. 3D Print Med..

[11] Ghosh R.M., Mascio C.E., Rome J.J., Jolley M.A., Whitehead K.K. (2021). Use of Virtual Reality for Hybrid Closure of Multiple Ventricular Septal Defects. JACC Case Rep..

[12] Krasemann T., Branstetter J. (2021). Virtual Reality Treatment Planning for Congenital Heart Disease. JACC Case Rep..

[13] Salavitabar A., Dutro M., Zablah J.E. (2024). Virtual Reality Remote Collaboration for Preprocedural Planning of Complex Percutaneous Congenital Interventions: A Case Series. J. Soc. Cardiovasc. Angiogr. Interv..

